# Balancing ethics and statistics: machine learning facilitates highly accurate classification of mice according to their trait anxiety with reduced sample sizes

**DOI:** 10.1038/s41398-025-03546-6

**Published:** 2025-08-21

**Authors:** Johannes Miedema, Beat Lutz, Susanne Gerber, Irina Kovlyagina, Hristo Todorov

**Affiliations:** 1 Institute of Human Genetics, University Medical Center of the Johannes Gutenberg University Mainz, Mainz, Germany; 2 Institute of Physiological Chemistry, University Medical Center of the Johannes Gutenberg University Mainz, Mainz, Germany; 3 Leibniz Institute for Resilience Research (LIR), Mainz, Germany; 4 Institute for Quantitative and Computational Biosciences (IQCB), Mainz, Germany; 5 Institute of Immunology, University Medical Center of the Johannes Gutenberg University Mainz, Mainz, Germany

**Keywords:** Molecular neuroscience, Learning and memory, Neuroscience

## Abstract

Understanding how individual differences influence vulnerability to disease and responses to pharmacological treatments represents one of the main challenges in behavioral neuroscience. Nevertheless, inter-individual variability and sex-specific patterns have been long disregarded in preclinical studies of anxiety and stress disorders. Recently, we established a model of trait anxiety that leverages the heterogeneity of freezing responses following auditory aversive conditioning to cluster female and male mice into sustained and phasic endophenotypes. However, unsupervised clustering required larger sample sizes for robust results which is contradictory to animal welfare principles. Here, we pooled data from 470 animals to train and validate supervised machine learning (ML) models for classifying mice into sustained and phasic responders in a sex-specific manner. We observed high accuracy and generalizability of our predictive models to independent animal batches. In contrast to data-driven clustering, the performance of ML classifiers remained unaffected by sample size and modifications to the conditioning protocol. Therefore, ML-assisted techniques not only enhance robustness and replicability of behavioral phenotyping results but also promote the principle of reducing animal numbers in future studies.

## Introduction

In 1959, the book “The Principles of Humane Experimental Technique” by William Russell and Rex Burch formally introduced the 3R principles to minimize animal use and suffering [1]. The 3Rs stand for Replacement, Reduction, and Refinement. While the principle of Refinement, which focuses on improving animal welfare, is widely accepted, the concepts of Replacement (utilizing alternative experimental models) and Reduction (minimizing the number of experimental animals) have remained highly controversial within the scientific community [2–4].

The principle of Reduction in particular raises ethical dilemmas as it creates a conflict between maintaining scientific rigor and minimizing animal usage [4]. Statistically underpowered animal studies could lead to the misinterpretation of research results, especially in the presence of high biological variability often seen in biomedical research, which necessitates larger experimental groups for robust findings [5]. Therefore, the inherent conflict between biological variability and the aim of minimizing animal numbers poses significant obstacles to the implementation of the Reduction principle. This challenge is particularly pronounced in animal behavioral research where variability is notorious but, nevertheless, integral and translationally valuable [6–8].

Recently, we developed a novel mouse model of trait anxiety [9] that leverages inter-individual variability of conditioned freezing responses combined with unsupervised clustering to phenotype inbred mice according to their anxiety trait. We demonstrated high construct and face validities of our model and showed that sustained freezing during prolonged memory retrieval sessions is an anxiety-endophenotype behavioral marker in female and male mice. The applicability of our experimental pipeline to both sexes is an important advantage, which helps to reduce the number of surplus animals. Furthermore, we directly addressed the sex-bias in translational research where female subjects have been severely underrepresented in experimental designs [10, 11]. To foster the replicability of our findings and enable the transfer of our pipeline to independent laboratories, we also implemented an Rshiny tool that provides an easy-to-use and transparent implementation of our multivariate statistical analysis framework [12].

However, achieving robust clustering of animals required relatively large sample sizes (n = 30–40), which could potentially limit the applicability of our pipeline. This restriction makes it particularly challenging to implement our model in mechanistic studies that cannot accommodate such large numbers of animals simultaneously.

To overcome these limitations, our current study utilized behavioral measures from 470 animals that had previously undergone our behavioral paradigm. We pooled data from several independent experiments to develop and validate sex-specific supervised machine learning (ML) models capable of accurately classifying mice into sustained and phasic freezing phenotypes. Importantly, once models have been trained, their performance is independent of sample sizes in new animal cohorts. This approach thus not only enhances the replicability of future experiments but also aligns with the Reduction principle by enabling even single-animal-level classification.

## Results

### Obtaining labeled training data for supervised ML from multiple animal cohorts

**Fig. 1:**
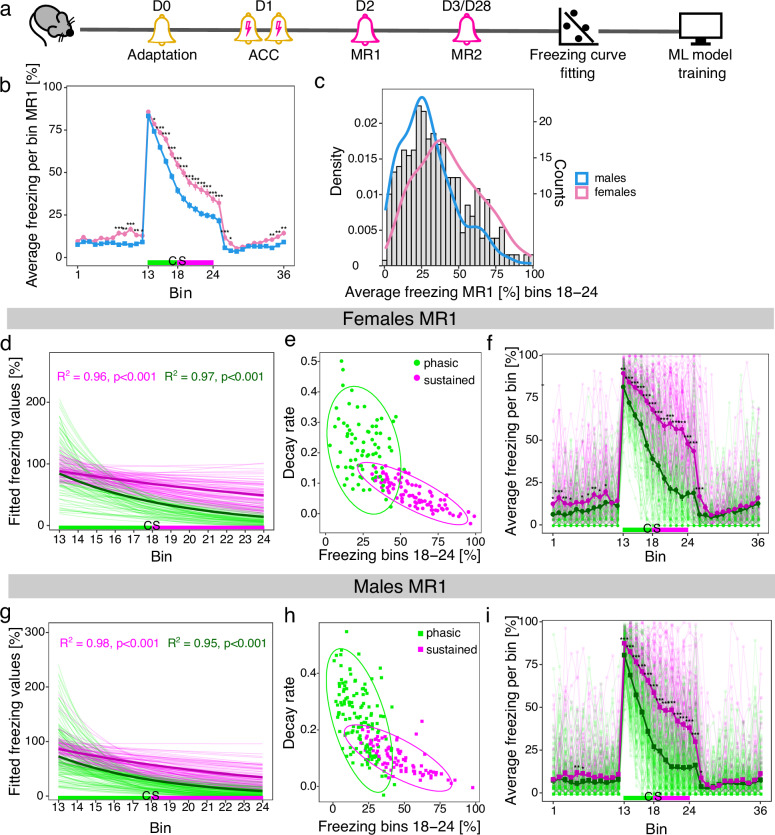
Clustering multiple pooled batches of female and male mice into sustained and phasic responders following auditory aversive conditioning. **a** Schematic representation of the study. **b** Average freezing responses of female compared to male mice during memory retrieval (MR) 1. **c** Histogram of average freezing responses during the last 3.5 min of cued stimulus (CS) presentation (bins 18–24) in MR1 for female and male mice. **d** Fitted freezing curves for sustained and phasic female responders during CS presentation in MR1. Fitted curves based on group average responses are indicated by thicker lines. R^2^-values correspond to the goodness-of-fit for the average fitted responses. **e** Bivariate scatter plots showing clustering of sustained and phasic female responders including 95% confidence ellipses around each cluster. **f** Freezing responses of sustained and phasic female responders during MR1. Average freezing responses are indicated by thicker lines whereas individual animal freezing curves are shown with lighter colors. **g** Fitted freezing curves for sustained and phasic male responders during CS presentation in MR1. Fitted curves based on group average responses are indicated by thicker lines. R^2^-values correspond to the goodness-of-fit for the average fitted responses. **h** Bivariate scatter plots showing clustering of sustained and phasic male responders including 95% confidence ellipses around each cluster. **i** Freezing responses of sustained and phasic male responders during MR1. Average freezing responses are indicated by thicker lines whereas individual animal freezing curves are shown with lighter colors. The time bins of cued stimulus (CS) presentation are colored in green (bins 13–17, first 2.5 min of CS presentation) and magenta (bins 18–24, last 3.5 min of CS presentation) in b, d, f, g, and i, corresponding to the phasic and sustained component of the freezing curve as described in Kovlyagina et al. [9] Values are shown as mean ± standard error of the mean in b, f and i. *** p < 0.001, **p < 0.01, *p < 0.05, linear mixed effect models followed by post-hoc comparisons of model means for female vs. male mice in b or sustained vs. phasic responders in f and i. N = 168 females (78 sustained and 90 phasic), n = 224 males (136 phasic and 88 sustained).

In our foundational work on establishing a behavioral pipeline to study trait anxiety in female and male inbred mice, we employed auditory aversive conditioning (AAC) protocols with prolonged memory retrieval (MR) sessions [9]. Extending conditioned stimulus (CS) presentation to 6 min instead of commonly used brief CS exposures of 30 s unlocked a range of freezing responses that allowed us to cluster animals into sustained and phasic responders based on their individual freezing behavior. To capture its dynamics, the freezing response during retrieval session was analyzed in 30-s time bins. We performed one MR session 24 h and the second MR session either 48 h or on day 28 after conditioning (Fig. 1a). Originally, each experimental batch was clustered separately using Gaussian mixture models (GMM). To obtain more reliable estimates of the population-level freezing response distributions, we therefore decided to pool data from multiple experimental cohorts together and repeat the clustering procedure (Fig. 1a). Consequently, we used data from 5 female and 8 male experimental batches (Supplementary Table 1).

In agreement with our previous results [9], we observed consistently higher freezing values in females compared to males in MR1 (Fig. 1b) and MR2 (Supplementary Fig. 1a). Furthermore, average sustained freezing (the last 3.5 min of CS presentation), identified as the main behavioral readout reflecting the animal’s anxiety trait [9], exhibited a right-skewed distribution towards lower values in male mice. In contrast, females showed a wider spread across the range of sustained freezing values (Fig. 1c, Supplementary Fig. 1b). These consistent sex-related differences in freezing behavior across multiple animal cohorts justified fitting separate models to assign female and male mice to distinct freezing phenotypes.

To this end, we employed our previously described strategy [9]. First, we utilized log-linear regression to model the freezing response of each animal during CS presentation (Fig. 1d, g; Supplementary Fig. 1c, f). Next, we employed GMM clustering using the intercept and slope (decay rate of freezing) of the fitted freezing curve as well as the average freezing response during the last 3.5 min of CS presentation, that we previously defined as sustained phase of the freezing response. Thus, we obtained two distinct subgroups of mice for each sex and MR (Fig. 1e, h; Supplementary Fig. 1d, g). Sustained responders were associated with significantly higher freezing during MR1 and MR2 for both sexes compared to phasic responders (Fig. 1f, i; Supplementary Fig. 1e, h). Differences were especially pronounced in the sustained phase of the freezing response during the last 3.5 min of CS presentation, where we already previously observed the highest inter-individual variability [9].

### Assessing clustering stability

**Fig. 2:**
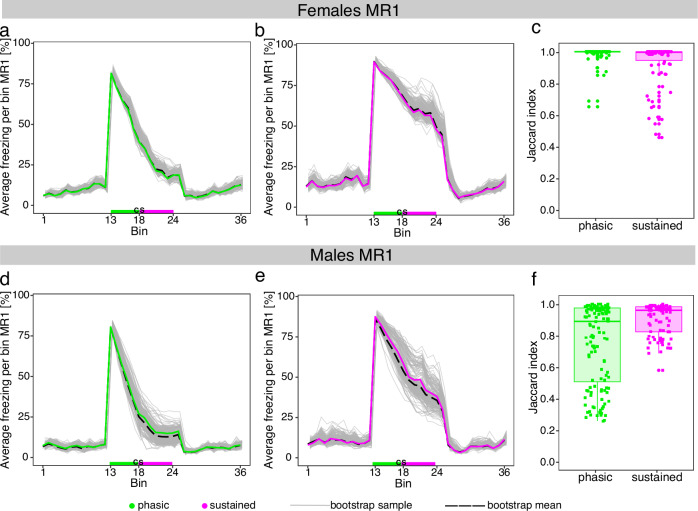
Bootstrap evaluation of clustering stability. Average freezing values for female phasic (**a**) and sustained responders (**b**) during memory retrieval (MR) 1. Average freezing values for male phasic (**d**) and sustained responders (**e**) during MR1. Grey lines correspond to average freezing responses of 200 bootstrap samples drawn from the original data with replacement separately for female and male animals. Dashed lines indicate the mean of all bootstrap samples for the respective experimental setup. Boxplots show Jaccard index values for each animal included in the bootstrap samples for female (**c**) and male (**f**) mice. The Jaccard index indicates the proportion of times an animal was assigned to its original cluster during the bootstrap sampling procedure. N phasic females = 78, n sustained females = 90, n phasic males = 136, n sustained males = 88.

While we pooled multiple animal cohorts in an effort to reliably model the population-level freezing response distributions, our clustering into sustained and phasic responders could still be influenced by sampling error. Therefore, we employed a bootstrap sampling procedure to ascertain the validity and robustness of our clustering results. To this end, we drew 200 random samples with replacement for female and male mice at MR1 and MR2 (see *Methods*) and compared clusters to the original phenotype assignment. Notably, we observed highly similar average freezing curves for sustained and phasic female and male mice in the bootstrap samples at MR1 (Fig. 2a, b, d, e) and MR2 (Supplementary Fig. 2a, b, d, e). Furthermore, average freezing remained higher for sustained compared to phasic responders even in the bootstrap samples. Next, we calculated the Jaccard index (see *Methods*) to investigate how often each animal retained its original cluster assignment in the bootstrap samples. We observed highly robust clustering results for female sustained and phasic responders at both MR1 (Fig. 2c) and MR2 (Supplementary Fig. 2c) as indicated by median Jaccard index values higher than 0.99. In contrast, we detected a higher variability and slightly lower median Jaccard index values for males compared to females at MR1 (Fig. 2f) and MR2 (Supplementary Fig. 2f). However, at the median, male animals also retained their original cluster assignment in more than 89% of the cases. These results clearly indicated that our animal cohorts reliably reflect population-level freezing distributions and clustering into sustained and phasic responders remains robust towards sampling errors for both female and male mice.

### Training supervised ML models to assign mice to sustained and phasic freezing phenotypes

Having demonstrated that pooling multiple experimental cohorts together results in stable clusters, we proceeded to use these labeled data to train supervised ML models. Notably, assigning mice to sustained and phasic phenotypes using ML classifiers trained on this larger dataset should produce more stable and reliable results in contrast to de novo clustering experimental batches separately in new experiments that could still be influenced by the sample size.

For model training, we utilized several well-established ML algorithms including support vector machines (SVM), logistic regression, linear discriminant analysis (LDA) and random forests. Since we applied our clustering procedure separately to each sex and MR, we trained individual ML models for each distinct experimental set-up. To evaluate the performance of the ML models, we split the labeled datasets into training and test sets with a ratio of 70:30%, respectively. However, we observed high variances between different training iterations, which is likely due to the limited sample sizes of the training sets. To address this issue, we performed 1000 iterations of randomly partitioning the dataset into training and test splits, which is a method known as Monte Carlo cross-validation [13]. Thus, we aimed at obtaining more robust estimates of each ML model’s performance by averaging the respective performance metric over all cross-validation iterations.

**Fig. 3:**
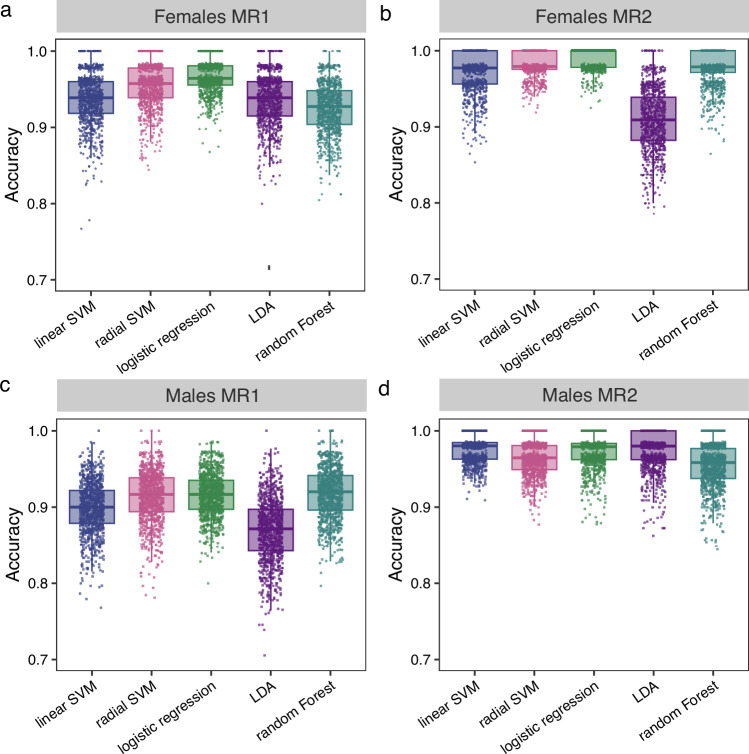
Accuracy of machine learning models to assign female and male mice to sustained and phasic freezing phenotypes. **a–d** Boxplots showing accuracies of different machine learning models to classify female and male mice into sustained and phasic phenotypes during memory retrieval (MR) 1 or 2. SVM: support vector machine, LDA: linear discriminant analysis. Model accuracies were calculated by subsetting the data in random training and test splits with a ratio of 70:30% over 1000 iterations. In each iteration the model was trained on the training data set; the respective accuracy was calculated on the test data set. N = 168 females, n = 224 males for MR1; n = 144 females, n = 180 males for MR2.

Remarkably, all ML models yielded high average accuracies exceeding 90% for each experimental scenario. Interestingly, ML models trained on data from female animals resulted in higher average accuracies than models trained on male animals for both MR sessions (Fig. 3a–d). Furthermore, models trained with MR2 datasets showed higher average accuracies than those trained with MR1 datasets for both sexes. Notably, for all scenarios except for male animals at MR1, several iterations reached perfect classification accuracies of 100%. For both female MR1 and MR2 training datasets, logistic regression yielded the highest average accuracy (Supplementary Table 2). For male animals, the random forest model and the SVM model with a linear kernel showed the highest average accuracy for MR1 and MR2, respectively. Based on this evaluation, we selected the best performing ML models (Table 1) and subsequently trained them using the complete respective dataset for further use in our study.

**Table 1:** Optimal supervised ML models for classifying female and male mice into sustained and phasic phenotypes for memory retrieval (MR) 1 and MR2.

Sex	Retrieval	Best performing model
Females	MR1	logistic regression
Females	MR2	logistic regression
Males	MR1	random forest
Males	MR2	SVM

The optimal model for each scenario was selected based on performance criteria including accuracy, specificity and sensitivity.

### Performance of ML models on independent animal batches

**Fig. 4:**
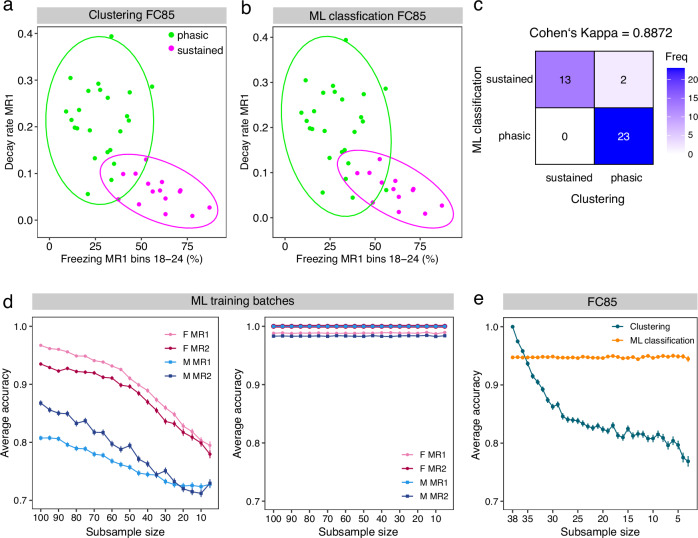
Validation of ML classification models on independent data. **a** Bivariate scatter plot showing Gaussian mixture model clustering results for the independent batch of female animals FC85 that was not included in training the supervised ML models. **b** Bivariate plot showing classification of FC85 into sustained and phasic responders using the pre-trained ML model. Individual data points including 95% confidence ellipses are shown. **c** Confusion matrix showing the concordance of clustering and ML classification results for FC85. **d** Average accuracies for subsamples of different sizes for clustering (left) compared to ML classification (right) based on the female and male batches of animals used for ML model training. **e** Average accuracies for subsamples of different sizes for clustering compared to ML classification based on FC85. Data are shown as mean ± standard error of the mean in (**d**) and (**e**). n = 38 in (**a**), (**b**), (**c**).

To assess whether the performance of our ML classifiers generalizes to independent data from different mouse lines, we used a batch (FC85) of 38 female Arc-nuGFP animals (Methods) that was not included in the pooled clustering and ML model training steps. FC85 females were subjected to our standard AAC pipeline and MRs were performed on day 2 and day 39. Subsequently, we used individual freezing parameters to assign mice to a sustained or phasic phenotype employing both our original clustering procedure and our supervised ML models. Notably, we observed an almost perfect agreement between both approaches for MR1 (Fig. 4a–c) and MR2 (Supplementary Fig. 3a–c) as also indicated by high Cohen’s kappa values greater than 0.885 (Fig. 4c, Supplementary Fig. 3c). Indeed, only two animals for both MR1 and MR2, respectively, were assigned to a different group using our ML classifier compared to standard GMM clustering.

While clustering and classification results were highly concordant, one significant advantage of ML models is that once they have been trained using a sufficiently large data set, their classification performance on new data is independent of sample size. In contrast, clustering results can quickly become unstable with small samples when de novo clustering is performed each time in independent animal cohorts. To further investigate this phenomenon empirically, we employed a subsampling procedure. In 1000 iterations, we drew subsamples of the female and male mice used for ML model training of varying sizes starting from 100 animals and going down to 5 animals. For each subsample, we then clustered animals into sustained and phasic responders and compared the average accuracy to the clustering results obtained with the total animal population. We observed a substantial drop in accuracy as subsample sizes decreased for both female and male animals in MR1 and MR2 (Fig. 4d, left). Next, we applied the same strategy with our pre-trained ML models. The performance remained unaffected by sample size as indicated by the uniformly high accuracy values (Fig. 4d, right). To address the bias that we tested the ML models on the data that was used for training, we performed the subsampling procedure with the independent batch (FC85) with subsample sizes ranging from 38 to 3 animals. Again, we observed a substantial decline in clustering accuracy for smaller samples. In contrast, the ML classifier maintained uniformly high accuracy around 0.95 across all sample sizes (Fig. 4e). These results clearly demonstrated the advantage of using pre-trained ML models to assign animals to sustained and phasic responders and highlighted the potential for substantial reduction of sample sizes in future experiments, supporting the 3R principles in animal research.

### ML classification into sustained and phasic responders remains robust towards AAC protocol modifications and batch variability

**Fig. 5:**
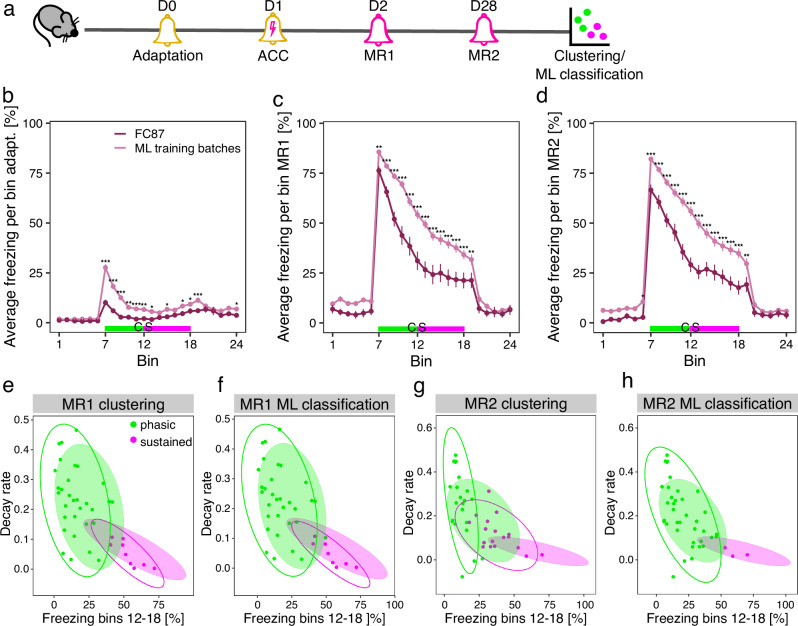
Effect of batch variability and AAC protocol modifications on clustering and ML classification. **a** Schematic representation of the experimental pipeline for the FC87 batch of female mice. Average freezing of female animals from the independent batch FC87 compared to female mice used for ML model training during adaptation (**b**), memory retrieval (MR) 1 (**c**) and MR2 (**d**). Data are shown as mean ± standard error of the mean. *** p < 0.001, **p < 0.01, *p < 0.05, linear mixed effect models followed by post-hoc comparisons of model means. The time bins of cued stimulus (CS) presentation are colored in green (first 2.5 min of CS presentation) and magenta (last 3.5 min of CS presentation) in (b)–(d), corresponding to the phasic and sustained component of the freezing curve as described in Kovlyagina et al. [9]. Due to the shortened pipeline for FC87, bins 7–18 (CS presentation) correspond to bins 13–24 for the ML training batches. **e**–**h** Bivariate scatter plots showing clustering and ML classification results for FC87 females. Data are shown as individual values and 95% confidence ellipses. The solid-color confidence ellipses were calculated based on the batches of female mice used for ML model training. N = 40 females in FC87, n = 166 for MR1 and n = 142 in the ML training batches. AAC auditory aversive conditioning.

In our original AAC protocol, we had two training sessions that were performed within 5 h of each other. To assess whether a single training session could still induce inter-individual variability and similar group-level freezing responses, we modified the conditioning protocol and tested it on a batch of 40 female mice (FC87, Fig. 5a).

Average freezing behavior of FC87 females was consistently lower at MR1 (Fig. 5c) and MR2 (Fig. 5d) compared to the ML training batches. Notably, we also observed significantly lower freezing on the adaptation day (Fig. 5b), suggesting that not only the AAC protocol modifications but also batch-specific variability influenced the freezing behavior of FC87 females.

To investigate the impact of these variations in our phenotyping procedure, we applied our standard clustering methods as well as our supervised ML classifiers to assign FC87 female mice into sustained and phasic responders. Interestingly, we observed high concordance of phenotyping results at MR1 with only one animal allocated differently between both approaches (Fig. 5e, f). However, clustering and ML classification differed drastically at MR2 (Fig. 5g, h). While batch-specific clustering produced two balanced groups, ML classification only assigned three animals to the sustained group. To further explore these discrepancies, we overlayed the confidence ellipses for the sustained and phasic groups derived from the female batches used for training the ML model (Fig. 5e–h, solid color confidence ellipses). FC87 batch-specific clustering at MR1 displayed similar distributions to the ML training batches. However, at MR2, FC87 clusters deviated substantially from the ML training batches, thereby highlighting the disparity between clustering and ML classification.

These results clearly demonstrated the benefits of using pre-trained supervised ML models for classifying animals into sustained and phasic responders. In the presence of protocol modifications or batch variability that cause shifts in the freezing response distributions, our ML classifiers provide robust assignment of mice to phenotypes that remain comparable across multiple animal cohorts.

### R package

To facilitate a consistent and reproducible utilization of our methods in future studies, we developed an R package – anxiotraitR available at https://github.com/johannesmiedema/anxiotraitR. The package takes freezing data obtained from our behavioral pipeline [9] as input and uses our best performing ML classifiers depending on sex and MR (Table 1) to allocate animals to sustained and phasic responders. Furthermore, we implemented the option to include additional data if they become available and use them to retrain the ML classifiers. This feature enables the continuous improvement and further refinement of our proposed models.

## Discussion

Resolving latent behavioral phenotypes remains a challenging task in animal studies, hampered by substantial variability in individual responses and high dimensionality of the data. The bigger sample sizes that such analyses inherently require are, however, in conflict with animal welfare principles. In our current study, we trained supervised ML models using pooled data from multiple cohorts to classify mice to sustained and phasic freezing phenotypes in a sex-specific manner. Our models showed high classification accuracy for independent data sets and their performance remained robust to modifications of the experimental pipeline.

Behavioral neuroscience studies typically utilize animals with identical genetic background. Although inbred lines are expected to minimize phenotypic variation, significant inter-individual differences in trait expression persist [8, 14–16]. Particularly in the context of translational research of fear and anxiety disorders, highly variable unconditioned and conditioned responses to threats have been reported [6, 17–19]. Failing to incorporate this factor in the research design and the analysis of behavioral data may negatively impact statistical power and replicability of results [5, 20]. In our previous study, we aimed at developing a translationally-relevant animal model of trait anxiety [9]. To this end, we harnessed the inter-individual variability in conditioned freezing responses to assign animals to distinct anxiety endophenotypes – sustained and phasic responders. Our model not only more accurately captures the variation in disease susceptibility and symptomatology of behavioral pathologies in humans [21–23] but also offers advantages from a statistical point of view. Modelling the spectrum of freezing responses as a systematic factor rather than random noise and identifying more homogenous subgroups in the general population of animals might offer an increased sensitivity to detect the effect of experimental manipulations or pharmacological treatments [20].

In addition to inter-individual variability of behavioral responses, high dimensionality of the data presents another major challenge in animal behavioral research. The advent of automatic tracking systems has enabled continuous monitoring of animals and the quantification of a vast number of behavioral variables with an unprecedented resolution [24, 25]. Data-driven approaches then rely on sophisticated deep learning architectures to elucidate distinct phenotypes or extract meaningful patterns from the multi-dimensional feature space [26–29]. However, training highly complex models based on limited sample sizes poses the risk of overfitting. Furthermore, increasing the number of measurements requires multiple testing adjustments to reduce the likelihood of false positive results. To circumvent the issue of multiple comparisons, von Zeigler and colleagues recently developed a technique called behavioral flow analysis [30]. This method combines clustering, supervised learning and dimensionality reduction to create a single composite measure for each animal based on multiple behavioral readouts. The authors argue that this approach increases statistical power to detect treatment effects of pharmacological or experimental interventions. However, as with other purely data-driven approaches, interpretation of model outputs is not trivial. Moreover, using non-linear dimensionality reduction techniques such as UMAP [31] prohibits correlating the composite score to the original behavioral domains.

In contrast, we employed a conceptually different approach to resolving latent freezing phenotypes. Rather than exclusively relying on a data-driven strategy, we grounded our experimental pipeline in a well-established theoretical framework: the threat imminence continuum theory [32]. Consequently, we postulated that the individuality of freezing responses reflects the animal’s subjective perception of a threat along the imminence continuum. Correlation with performance in approach-avoidance tests demonstrated that freezing serves as a robust and replicable behavioral marker of mouse trait anxiety that can be leveraged to assign inbred mice to distinct anxiety endophenotypes [9]. However, our original approach relied on clustering experimental batches separately which required bigger sample sizes to obtain robust clustering results. Using a Monte-Carlo subsampling simulation, we demonstrated that model accuracy substantially dropped for smaller sample sizes (Fig. 4d, e). Furthermore, modifying our conditioning protocol to include one instead of two training sessions led to a significant decrease in the average freezing response. Subsequently, batch-specific clustering deviated substantially from previous results (Fig. 5e–h). Thus, purely data-driven clustering might reduce comparability and generalizability of our inferred phenotypes across independent cohorts. We utilized GMM clustering where results might be influenced by small sample sizes in several ways. For instance, parameter estimates of the multivariate normal distributions underlying each cluster have a higher variance and therefore might be statistically less reliable. Furthermore, individual data points have a larger influence on parameter estimation in smaller samples, making results more sensitive to outliers [33]. In contrast, by using a bootstrapping approach, we demonstrated that clustering results obtained from pooling multiple animal batches remained robust (Fig. 2). Training supervised ML models on these pooled data offers key advantages for future use of our experimental pipeline. For example, the inferred sustained and phasic groups are generalizable and comparable across independent experiments. More importantly, our ML classifiers only depend on group sizes in the initial training step and once trained, they retain high classification accuracy irrespective of sample size in new cohorts thus promoting the Reduction principle in animal research (Fig. 4d, e). Future experiments adopting our experimental pipeline can therefore focus on targeted phenotyping and characterization even of single animals. By comparing freezing values from small samples against our previously generated reference data, we can gain insights into the magnitude of the sustained or phasic phenotypes by revealing where each animal is placed in the population-level response distribution. Furthermore, our R package anxiotraitR implements the option to retrain the ML models whenever newly generated data exceed the original dataset used for ML training, thus ensuring continuous improvement of our models.

Another important advantage of our study is the sex-specificity of our ML models to assign mice to sustained and phasic freezing phenotypes. While women are more susceptible to anxiety and mood disorders than men [34, 35], animal studies in neuroscience have continuously excluded sex as a biological variable, mainly focusing on males [36, 37]. In our study, females consistently demonstrated increased freezing during MR sessions even when we pooled independent animal cohorts obtained from different breeding facilities. These systematic sex-related differences in the multivariate freezing response distributions were also reflected by distinct ML algorithms providing the best performance for females compared to males (Table 1). The ability to classify both female and male mice according to their trait anxiety enhances the translational value of our model as this feature facilitates the study of sex-specific mechanisms of behavioral pathologies or efficacy of therapeutic interventions.

However, our testing and computational pipeline was developed primarily using C57BL/6J mice in a single laboratory. While we included animals from three different commercial providers and replicated key findings in independent transgenic mouse lines bred in-house, the generalizability of our approach to other laboratories and mouse strains remains to be confirmed in future studies. To enable independent research groups to directly apply and test our methodology, we have made both the experimental procedures [9] and computational tools [12] openly available. Furthermore, we have implemented the option to retrain and improve our ML models as new, more diverse data sets become available.

In summary, our study represents a significant advancement in the application of ML techniques to reduce animal cohort sizes in future research. By combining a validated behavioral testing pipeline with a sex-specific ML classifier we offer a comprehensive toolbox for mechanistic and pharmacological studies of trait anxiety. Our approach not only enhances the precision of phenotyping but also supports ethical considerations in animal research by enabling robust analyses with fewer animals.

## Material and methods

### Animals

All experiments were conducted following the European Community’s Council Directive of 22 September 2010 (2010/63EU) and approved by the Landesuntersuchungsamt of the State Rhineland-Palatinate, Germany; file numbers of ethical approvals: 23 177-07/G16-1-085, 23 177-07/G17-1-028, and 23 177-07/G21-17-028. 8–9-week-old C57BL/6J mice were purchased from the Charles River or Janvier breeding facilities and allowed to habituate to the new facility for at least 3 weeks. For performance generalizability evaluation, data from the reporter mouse line Arc-nuGFP were used (batch FC85). Arc-nuGFP mice were generated by crossing B6.Cg-Tg(Arc-cre/ERT2)MRhn/CdnyJ mice (JAX Nr. 022357 [38]) with B6.129-Gt(ROSA)26Sortm5(CAG-Sun1/sfGFP)Nat/J mice [39]. The Arc-nuGFP batch was formed from the resulting double transgenic heterozygous mice. Mice were group-housed in temperature- and humidity-controlled rooms with a 12-h light-dark cycle. Water and food were provided ad libitum. Animals were single-housed for 7 days before starting behavioral experiments. All mice were at least 12 weeks old at the beginning of testing. Behavioral tests were conducted during the light phase.

### Auditory aversive conditioning (AAC)

The AAC paradigm was performed as described in our previous study [9] using an unpredictable conditioning protocol. Briefly, on day 0 (adaptation), mice were habituated to context A (cylinder wrapped in nontransparent paper, electric grid covered by the white plastic panel, fresh bedding was used for each mouse, chambers were cleaned between trials with water). Each mouse was placed in the cylinder and allowed to explore freely for 3 or 6 min. Afterwards, mice were presented with a Tone (6 min/10 kHz, 75 dB) and then left in the chambers for 3 or 6 more minutes. On the next day (day 1), mice underwent AAC in context B (transparent rectangular, electric grid not covered, chambers were cleaned between trials with 1% acetic acid). We employed an unpredictable conditioning protocol with a 2-min habituation, 4xCS (29 s, 9 s, 19 s, 15 s/10 kHz, 75 dB), ISI 30 s; each CS was co-terminated with 1 s 0.4 mA electric foot shock. After the last shock, mice were left in the chambers for 30 s. Training was performed again 5 h later with an altered CS order (14 s, 19 s, 9 s, 29 s). Memory retrieval sessions were performed in context A (cylinder wrapped in nontransparent paper, electric grid covered by the white plastic panel, fresh bedding was used for each mouse, chambers were cleaned between trials with water) following the same protocol from adaptation day. For adaptation, 0.5 s and for MRs, 1 s freezing thresholds were applied for final data analysis. While mice were not specifically habituated to experimenters, we did not observe a significant experimenter effect [9]. The AAC paradigm was facilitated using TSE Multi Conditioning chambers, series 256060 (TSE Systems GmbH, Bad Homburg, Germany). Animals were video recorded and tracked automatically offline using Ethovision 13 (Noldus, Wageningen, The Netherlands).

### Modified AAC training protocol

To understand the influence of the second training session on the freezing response, we modified the AAC paradigm by removing one training session and applying 6 foot shocks in the first training instead. We employed an unpredictable conditioning protocol with a 2-min habituation, 6xCS (29 s, 9 s, 19 s, 15 s, 14 s, 19 s/10 kHz, 75 dB), ISI 30 s; each CS was co-terminated with 1 s 0.4 mA electric foot shock. After the last shock, mice were left in the chambers for 30 s. Adaptation and memory retrieval sessions were performed according to standard protocols described above.

### Clustering mice into sustained and phasic freezers

Log-linear regression analysis to model individual animal’s freezing behavior and GMM clustering to separate mice into sustained and phasic responders was performed following the same procedure as described in detail in our previous study [9]. Briefly, we used log-linear regression to model the freezing behavior of each animal during CS presentation in MR sessions. We then used the intercept and slope of the fitted freezing curve together with the average freezing during the last 3.5 min of CS presentation to cluster animals into sustained and phasic phenotypes. Gaussian mixture model clustering was performed with the mclust [40] package v6.0.1. A total of 168 female mice from five different batches were pooled together for clustering following MR1. For MR2 analysis, 144 female animals from four different batches were used. Furthermore, 224 male mice from nine different batches were pooled together for clustering after MR1. For MR2 analysis, two batches were excluded due to missing MR2 freezing values, therefore 180 male animals from seven different batches were used. An overview of all animal batches is available in Supplementary Table 1.

### Bootstrap evaluation of clustering stability

To evaluate the validity and stability of our clustering approach, we applied a bootstrapping procedure as described by van der Goot and colleagues [20]. For each sex and MR, we drew 200 random samples with replacement with n equal to the size of the original sample. We then assessed the similarity of cluster composition between bootstrap samples and our original analysis by calculating the Jaccard index for each animal:

$${{Jaccard\; index}}_{{{mouse}}_{i}}=\frac{{number\; of\; times\; in\; original\; cluster}}{{total\; nuber\; of\; boostrap\; samples\; including\;}{{mouse}}_{i}}$$

The overall cluster stability was then determined by comparing median Jaccard indices for each cluster.

### ML model training

Labelled freezing data obtained from our previously described GMM clustering were used to train ML-models separately for female and male animals. To assign mice to sustained and phasic phenotypes using MR1 or MR2 data, the following binary classification algorithms were employed: support vector machines (SVM) with linear and radial kernels, logistic regression, linear discriminant analysis (LDA) and random forests, using the R packages e1071 v1.7.14, base v4.3.2, MASS [41] v7.3.60.0.1 and randomForest [42] v4.7.1.1, respectively. The intercept and regression coefficient of the fitted freezing curve of each animal from the log-linear regression model as well as the average freezing during the last 3.5 min of CS presentation in the MR sessions were used as input features for the ML algorithms.

### ML model performance evaluation

To evaluate the performance of different ML-models, the data were split randomly into a training and test set with a ratio of 70:30%. Performance metrics such as accuracy, sensitivity, specificity and F1 were then calculated using the caret [43] package v6.0.94. AUROC measurements were obtained using the pROC [44] package v1.18.5. With true positives (TN), false positives (FP), true negatives (TN) and false negatives (FN), the accuracy, sensitivity, specificity and F1-scores were calculated using the following formulas:

$${Accuracy}=\frac{{TP}+{TN}}{{TP}+{TN}+{FP}+{FN}}$$

$${Sensitivity}=\frac{{TP}}{{TP}+{FN}}$$

$${Specificity}=\,\frac{{TN}}{{TN}+{FP}}$$

$$F1=\,\frac{{TP}}{{TP}+0.5* ({FP}+{FN})}$$

We observed high variance between the metrics in different iterations of the training and test split. Thus, we employed Monte Carlo cross-validation [13] with 1000 iterations to obtain more stable estimates of the performance metrics. This method can be used to validate models that have been trained on datasets with small samples sizes. The final estimate for the respective metric was calculated as the mean over the 1000 iterations:

$${metric}=\frac{1}{1000}\,\mathop{\sum }\limits_{i=1}^{1000}{{metric}}_{i}$$

### R package implementation

To facilitate reproducibility and ease-of-use, we incorporated the best performing predictive models for each experimental set-up (Table 1) in an R package anxiotraitR, available at: https://github.com/johannesmiedema/anxiotraitR. For the implementation and development of anxiotraitR, the packages roxygen2 v7.3.1 and devtools v2.4.5 were used.

### Testing performance of ML models on independent batches

To evaluate our ML classifiers’ performance on independent data, we used two batches (FC85 and FC87) of female animals that were not included in training the ML models. To directly compare the classification results with the original clustering approach, we first clustered animals from each batch separately, using log-linear regression and GMM clustering as described above. Then we produced confusion matrices to assess the agreement of the clustering and ML classification results using the caret [43] package v6.0.94. Furthermore, we calculated Cohen’s kappa as a measure of concordance using DescTools v0.99.54.

### Evaluating the effect of sample size on clustering results

We employed a Monte-Carlo subsampling strategy to demonstrate the effect of sample size on clustering results. In 1000 iterations, we drew random subsamples for each sex and MR session from the labeled dataset used for ML-model training. Subsequently, for each subsampling step, the accuracy of clustering results in the random subsample versus the clustering results using the whole dataset was calculated. The mean accuracy over all iterations for each subsample size was then visualized with ggplot2 v3.4.4. Furthermore, we performed the same procedure with the independent batch FC85, using the clustering results with all animals from this batch as the ground truth for calculating clustering accuracy. We also performed classification on the respective subsamples using our pre-trained supervised ML models and calculated classification accuracy over all subsampling iterations.

### Statistical analysis

Group comparisons of average freezing values over time between sustained and phasic freezers or male and female animals were facilitated using linear mixed effects models as implemented in the lme4 [45] package v1.1.35.1. Pairwise comparisons of model means were performed using the emmeans package v1.10.0. Two-tailed p-values < = 0.05 were considered statistically significant. Results were visualized with ggplot2 v3.4.4.

## Supplementary information

Supplementary Figures and Tables

## Data Availability

The data used for model training as well as the corresponding code are included in the anxiotraitR R package, available on GitHub: https://github.com/johannesmiedema/anxiotraitR.

## References

[1] Russell WMS, Burch RL. The principles of humane experimental technique. London: Methuen; 1959.

[2] Aerts L, Miccoli B, Delahanty A, Witters H, Verstraelen S, De Strooper B, et al. Do we still need animals? Surveying the role of animal-free models in Alzheimer’s and Parkinson’s disease research. Embo J. 2022;41:e110002 10.15252/embj.2021110002. 35199384 10.15252/embj.2021110002 PMC8922267

[3] Genzel L, Adan R, Berns A, van den Beucken J, Blokland A, Boddeke E, et al. How the COVID-19 pandemic highlights the necessity of animal research. Curr Biol. 2020;30:R1014–r8. 10.1016/j.cub.2020.08.030. 32961149 10.1016/j.cub.2020.08.030 PMC7416712

[4] Würbel H. More than 3Rs: the importance of scientific validity for harm-benefit analysis of animal research. Lab Anim. 2017;46:164–6. 10.1038/laban.1220. 10.1038/laban.1220 28328898

[5] Voelkl B, Altman NS, Forsman A, Forstmeier W, Gurevitch J, Jaric I, et al. Reproducibility of animal research in light of biological variation. Nat Rev Neurosci. 2020;21:384–93. 10.1038/s41583-020-0313-3. 32488205 10.1038/s41583-020-0313-3

[6] Dopfel D, Perez PD, Verbitsky A, Bravo-Rivera H, Ma Y, Quirk GJ, et al. Individual variability in behavior and functional networks predicts vulnerability using an animal model of PTSD. Nat Commun. 2019;10:2372 10.1038/s41467-019-09926-z. 31147546 10.1038/s41467-019-09926-z PMC6543038

[7] Honegger K, de Bivort B. Stochasticity, individuality and behavior. Curr Biol. 2018;28:R8–r12. 10.1016/j.cub.2017.11.058. 29316423 10.1016/j.cub.2017.11.058

[8] Laskowski KL, Bierbach D, Jolles JW, Doran C, Wolf M. The emergence and development of behavioral individuality in clonal fish. Nat Commun. 2022;13:6419 10.1038/s41467-022-34113-y. 36307437 10.1038/s41467-022-34113-y PMC9616841

[9] Kovlyagina I, Wierczeiko A, Todorov H, Jacobi E, Tevosian M, von Engelhardt J, et al. Leveraging interindividual variability in threat conditioning of inbred mice to model trait anxiety. PLOS Biol. 2024;22:e3002642 10.1371/journal.pbio.3002642. 38805548 10.1371/journal.pbio.3002642 PMC11161093

[10] Will TR, Proaño SB, Thomas AM, Kunz LM, Thompson KC, Ginnari LA, et al. Problems and progress regarding sex bias and omission in neuroscience research. eNeuro. 2017;4:ENEURO.0278–17.2017. 10.1523/eneuro.0278-17.2017. 29134192 10.1523/ENEURO.0278-17.2017 PMC5677705

[11] Woitowich NC, Beery A, Woodruff T. A 10-year follow-up study of sex inclusion in the biological sciences. Elife. 2020;9:e56344 10.7554/eLife.56344. 32513386 10.7554/eLife.56344 PMC7282816

[12] Busch AM, Kovlyagina I, Lutz B, Todorov H, Gerber S. beeRapp: an R shiny app for automated high-throughput explorative analysis of multivariate behavioral data. Bioinform Adv. 2022;2:vbac082 10.1093/bioadv/vbac082. 36699414 10.1093/bioadv/vbac082 PMC9710645

[13] Shan G. Monte Carlo cross-validation for a study with binary outcome and limited sample size. BMC Med Inform Decis Mak. 2022;22:270 10.1186/s12911-022-02016-z. 36253749 10.1186/s12911-022-02016-z PMC9578204

[14] Freund J, Brandmaier AM, Lewejohann L, Kirste I, Kritzler M, Krüger A, et al. Emergence of individuality in genetically identical mice. Science. 2013;340:756–9. 10.1126/science.1235294. 23661762 10.1126/science.1235294

[15] Hager T, Jansen RF, Pieneman AW, Manivannan SN, Golani I, van der Sluis S, et al. Display of individuality in avoidance behavior and risk assessment of inbred mice. Front Behav Neurosci. 2014;8:314 10.3389/fnbeh.2014.00314. 25278853 10.3389/fnbeh.2014.00314 PMC4165351

[16] Ali Nasser R, Harel Y, Stern S. Early-life experience reorganizes neuromodulatory regulation of stage-specific behavioral responses and individuality dimensions during development. eLife. 2023;12:e84312 10.7554/eLife.84312. 37195038 10.7554/eLife.84312 PMC10234630

[17] Bush DE, Sotres-Bayon F, LeDoux JE. Individual differences in fear: isolating fear reactivity and fear recovery phenotypes. J Trauma Stress. 2007;20:413–22. 10.1002/jts.20261. 17721971 10.1002/jts.20261

[18] Cohen H, Zohar J, Matar M. The relevance of differential response to trauma in an animal model of posttraumatic stress disorder. Biol Psychiatry. 2003;53:463–73. 10.1016/s0006-3223(02)01909-1. 12644351 10.1016/s0006-3223(02)01909-1

[19] Shumake J, Jones C, Auchter A, Monfils MH. Data-driven criteria to assess fear remission and phenotypic variability of extinction in rats. Philos Trans R Soc Lond B Biol Sci. 2018;373:20170035 10.1098/rstb.2017.0035. 29352033 10.1098/rstb.2017.0035 PMC5790832

[20] van der Goot MH, Kooij M, Stolte S, Baars A, Arndt SS, van Lith HA. Incorporating inter-individual variability in experimental design improves the quality of results of animal experiments. PLoS One. 2021;16:e0255521 10.1371/journal.pone.0255521. 34351958 10.1371/journal.pone.0255521 PMC8341614

[21] Al Jowf GI, Snijders C, Rutten BPF, de Nijs L, Eijssen LMT. The molecular biology of susceptibility to post-traumatic stress disorder: highlights of epigenetics and epigenomics. Int J Mol Sci. 2021;22:10743 10.3390/ijms221910743. 34639084 10.3390/ijms221910743 PMC8509551

[22] Chiu HTS, Low DCW, Chan AHT, Meiser-Stedman R. Relationship between anxiety sensitivity and post-traumatic stress symptoms in trauma-exposed adults: a meta-analysis. J Anxiety Disord. 2024;103:102857 10.1016/j.janxdis.2024.102857. 38507961 10.1016/j.janxdis.2024.102857

[23] Horn SR, Charney DS, Feder A. Understanding resilience: new approaches for preventing and treating PTSD. Exp Neurol. 2016;284:119–32. 10.1016/j.expneurol.2016.07.002. 27417856 10.1016/j.expneurol.2016.07.002

[24] Pereira TD, Shaevitz JW, Murthy M. Quantifying behavior to understand the brain. Nat Neurosci. 2020;23:1537–49. 10.1038/s41593-020-00734-z. 33169033 10.1038/s41593-020-00734-z PMC7780298

[25] von Ziegler L, Sturman O, Bohacek J. Big behavior: challenges and opportunities in a new era of deep behavior profiling. Neuropsychopharmacology. 2021;46:33–44. 10.1038/s41386-020-0751-7. 32599604 10.1038/s41386-020-0751-7 PMC7688651

[26] Berman GJ. Measuring behavior across scales. BMC Biol. 2018;16:23 10.1186/s12915-018-0494-7. 29475451 10.1186/s12915-018-0494-7 PMC5824583

[27] Datta SR, Anderson DJ, Branson K, Perona P, Leifer A. Computational neuroethology: a call to action. Neuron. 2019;104:11–24. 10.1016/j.neuron.2019.09.038. 31600508 10.1016/j.neuron.2019.09.038 PMC6981239

[28] Klibaite U, Kislin M, Verpeut JL, Bergeler S, Sun X, Shaevitz JW, et al. Deep phenotyping reveals movement phenotypes in mouse neurodevelopmental models. Mol Autism. 2022;13:12 10.1186/s13229-022-00492-8. 35279205 10.1186/s13229-022-00492-8 PMC8917660

[29] Mathis A, Mamidanna P, Cury KM, Abe T, Murthy VN, Mathis MW, et al. DeepLabCut: markerless pose estimation of user-defined body parts with deep learning. Nat Neurosci. 2018;21:1281–9. 10.1038/s41593-018-0209-y. 30127430 10.1038/s41593-018-0209-y

[30] von Ziegler LM, Roessler FK, Sturman O, Waag R, Privitera M, Duss SN, et al. Analysis of behavioral flow resolves latent phenotypes. Nat Methods. 2024;21:2376–87. 10.1038/s41592-024-02500-6. 39533008 10.1038/s41592-024-02500-6 PMC11621029

[31] McInnes L, Healy J, Saul N, Großberger L. UMAP: uniform manifold approximation and projection. J Open Source Softw. 2018;3:861 10.21105/joss.00861.

[32] Fanselow MS, Lester LS A functional behavioristic approach to aversively motivated behavior: Predatory imminence as a determinant of the topography of defensive behavior. Evolution and learning. Hillsdale, NJ, US: Lawrence Erlbaum Associates, Inc; 1988. pp. 185-212.

[33] Fraley C, Raftery AE. Model-based clustering, discriminant analysis, and density estimation. J Am Stat Assoc. 2002;97:611–31. 10.1198/016214502760047131.

[34] McLean CP, Asnaani A, Litz BT, Hofmann SG. Gender differences in anxiety disorders: prevalence, course of illness, comorbidity and burden of illness. J Psychiatr Res. 2011;45:1027–35. 10.1016/j.jpsychires.2011.03.006. 21439576 10.1016/j.jpsychires.2011.03.006 PMC3135672

[35] Seedat S, Scott KM, Angermeyer MC, Berglund P, Bromet EJ, Brugha TS, et al. Cross-national associations between gender and mental disorders in the World Health Organization World Mental Health Surveys. Arch Gen Psychiatry. 2009;66:785–95. 10.1001/archgenpsychiatry.2009.36. 19581570 10.1001/archgenpsychiatry.2009.36 PMC2810067

[36] Rechlin RK, Splinter TFL, Hodges TE, Albert AY, Galea LAM. An analysis of neuroscience and psychiatry papers published from 2009 and 2019 outlines opportunities for increasing discovery of sex differences. Nat Commun. 2022;13:2137 10.1038/s41467-022-29903-3. 35440664 10.1038/s41467-022-29903-3 PMC9018784

[37] Shansky RM. Are hormones a “female problem” for animal research? Science. 2019;364:825–6. 10.1126/science.aaw7570. 31147505 10.1126/science.aaw7570

[38] Denny CA, Kheirbek MA, Alba EL, Tanaka KF, Brachman RA, Laughman KB, et al. Hippocampal memory traces are differentially modulated by experience, time, and adult neurogenesis. Neuron. 2014;83:189–201. 10.1016/j.neuron.2014.05.018. 24991962 10.1016/j.neuron.2014.05.018 PMC4169172

[39] Mo A, Mukamel EA, Davis FP, Luo C, Henry GL, Picard S, et al. Epigenomic signatures of neuronal diversity in the mammalian brain. Neuron. 2015;86:1369–84. 10.1016/j.neuron.2015.05.018. 26087164 10.1016/j.neuron.2015.05.018 PMC4499463

[40] Scrucca L, Fraley C, Murphy TB, Raftery AE. Model-based clustering, classification, and density estimation using mclust in R: Chapman and Hall/CRC; 2023.

[41] Venables WN, Ripley BD. Modern Applied Statistics with S: Springer; Springer-Verlag New York, 2002.

[42] Liaw A, Wiener M. Classification and regression by randomForest. R N. 2002;2:18–22.

[43] Kuhn M. Building predictive models in R using the caret package. J Stat Softw. 2008;28:1–26. 10.18637/jss.v028.i05.27774042

[44] Robin X, Turck N, Hainard A, Tiberti N, Lisacek F, Sanchez J-C, et al. pROC: an open-source package for R and S+ to analyze and compare ROC curves. BMC Bioinforma. 2011;12:77 10.1186/1471-2105-12-77. 10.1186/1471-2105-12-77 PMC3068975 21414208

[45] Bates D, Mächler M, Bolker B, Walker S. Fitting linear mixed-effects models using lme4. J Stat Softw. 2015;67:1–48. 10.18637/jss.v067.i01.

