# Supplementary material for: Balancing ethics and statistics: machine learning facilitates highly accurate classification of mice according to their trait anxiety with reduced sample sizes
Source: Transl Psychiatry. 2025 Aug 21;15:304. doi: 10.1038/s41398-025-03546-6 (PMC12370989; doi:10.1038/s41398-025-03546-6)
Supplement: Supplementary file 1 — Supplementary Figures and Tables [file 41398_2025_3546_MOESM1_ESM.docx]

**Balancing ethics and statistics: Machine learning facilitates highly accurate classification of mice according to their trait anxiety with reduced sample sizes**

Johannes Miedema, Beat Lutz, Susanne Gerber, Irina Kovlyagina & Hristo Todorov


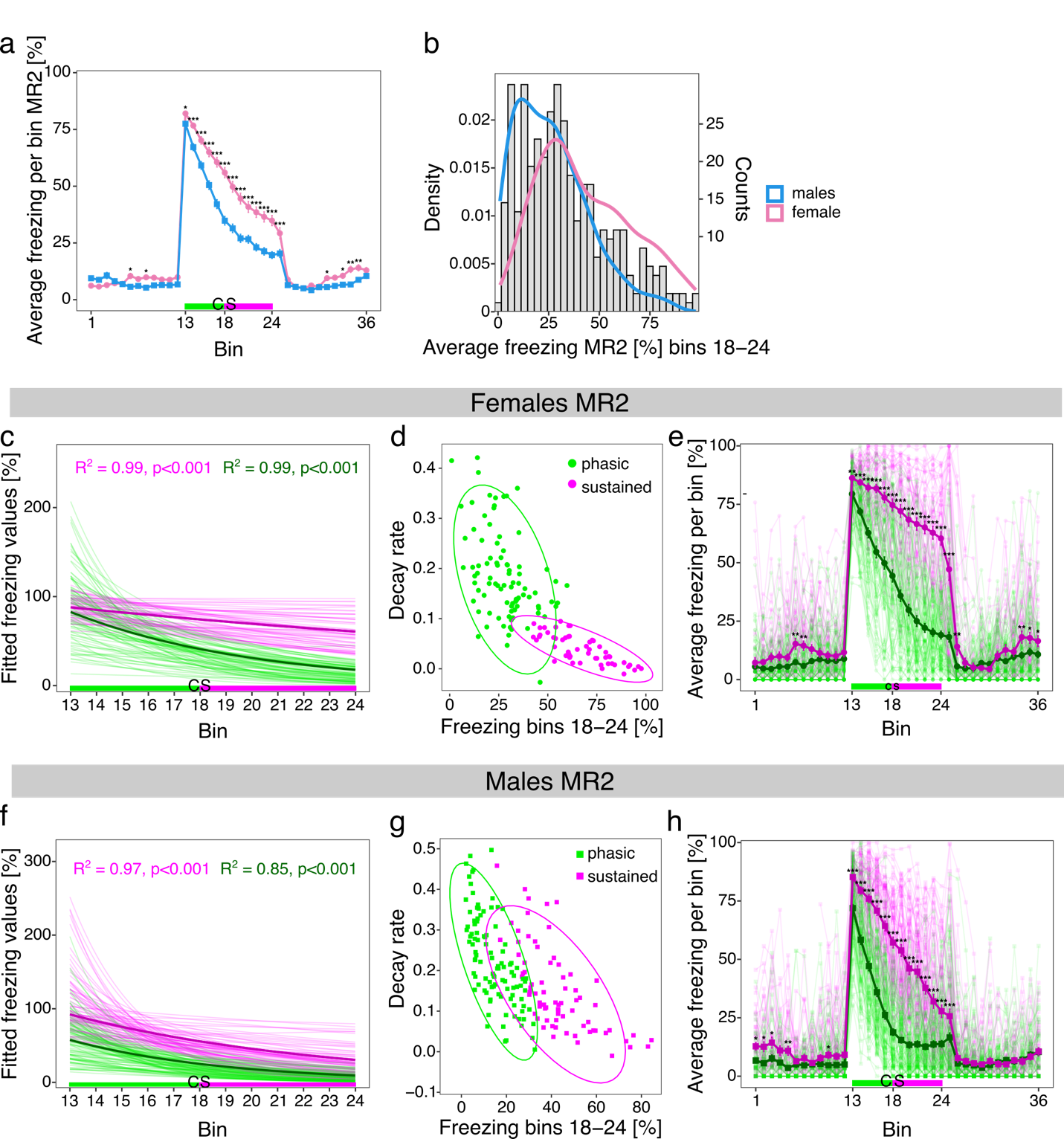


**Supplementary Figure 1. Clustering multiple batches of female and male mice into sustained and phasic responders following auditory aversive conditioning at memory retrieval (MR) 2.**

**a)** Average freezing responses of female compared to male mice during MR2. **b)** Histogram of average freezing responses during the last 3.5 min of cued stimulus (CS) presentation (bins 18-24) in MR2 for female and male mice. **c)** Fitted freezing curves for sustained and phasic female responders during CS presentation in MR2. Fitted curves based on average responses are indicated by thicker lines. R^2^-values correspond to the goodness-of-fit for the average fitted responses. **d)** Bivariate scatter plots showing clustering of sustained and phasic female responders including 95% confidence ellipses around each cluster. **e)** Freezing responses of sustained and phasic female responders during MR2. Average freezing responses are indicated by thicker lines whereas individual animal freezing curves are shown with lighter colors. **f)** Fitted freezing curves for sustained and phasic male responders during CS presentation in MR1. Fitted curves based on average responses are indicated by thicker lines. R^2^-values correspond to the goodness-of-fit for the average fitted responses. **g)** Bivariate scatter plots showing clustering of sustained and phasic male responders including 95% confidence ellipses around each cluster. **h)** Freezing responses of sustained and phasic male responders during MR2. Average freezing responses are indicated by thicker lines whereas individual animal freezing curves are shown with lighter colors. The time bins of cued stimulus (CS) presentation are colored in green (bins 13-17, first 2.5 min of CS presentation) and magenta (bins 18-24, last 3.5 of CS presentation) in a, c, e, f, and h, corresponding to the phasic and sustained component of the freezing curve as described in Kovlyagina et al. [1]. Values are shown as mean ± standard error of the mean in a, e and h. *** p<0.001, **p<0.01, *p<0.05, linear mixed effect models followed by post-hoc comparisons of model means for female vs. male mice in a or sustained vs. phasic responders in e and h. n = 144 for females (89 phasic and 55 sustained), n = 180 for males (105 phasic and 75 sustained).


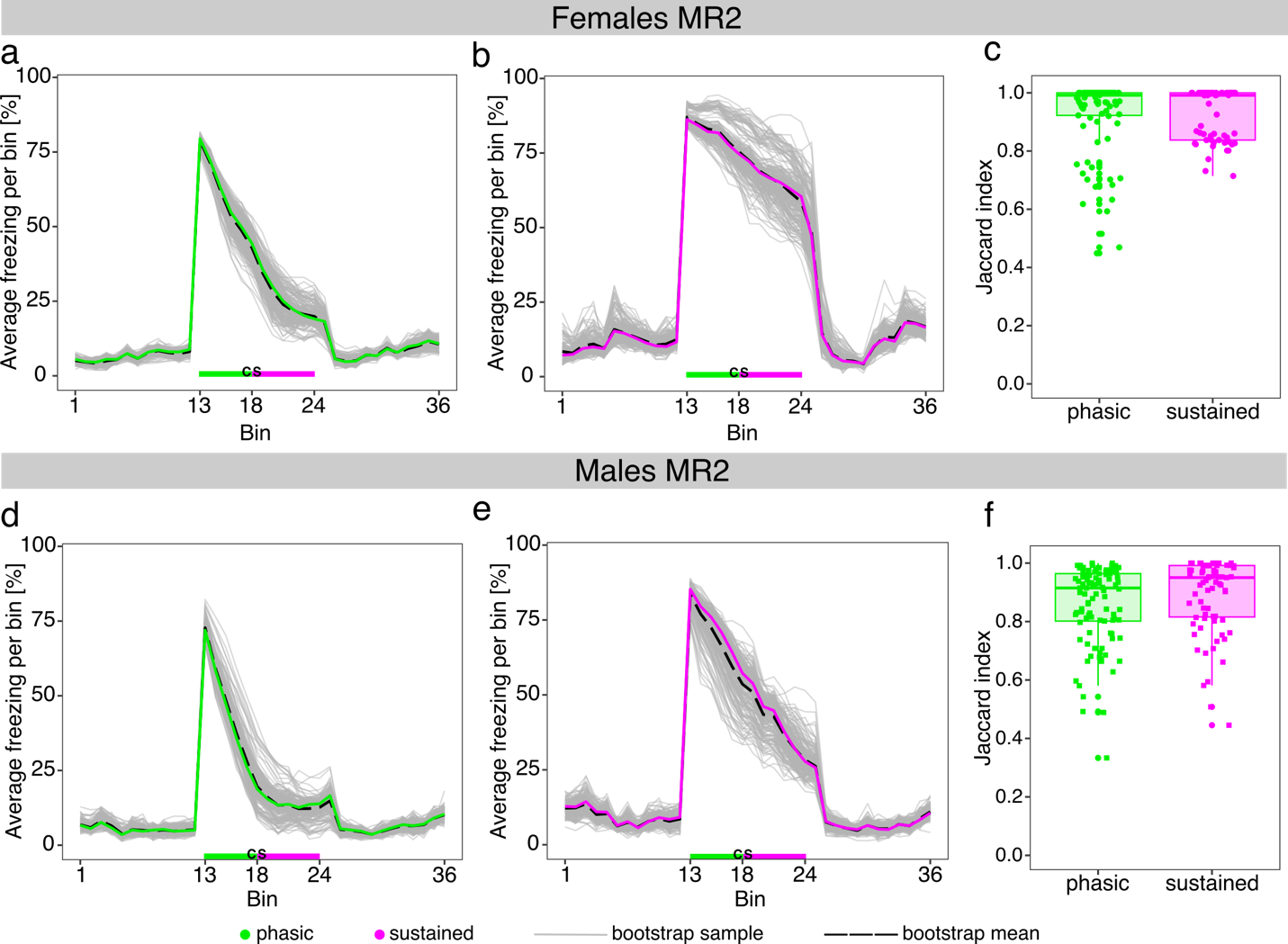


**Supplementary Figure 2. Bootstrap evaluation of clustering stability for memory retrieval (MR) 2.** Average freezing values for female phasic **(a)** and sustained responders **(b)** during MR 2**.** Average freezing values for male phasic **(d)** and sustained responders **(e)** during MR2. Grey lines correspond to average freezing responses of 200 bootstrap samples drawn from the original data with replacement separately for female and male animals. Dashed lines indicate the mean of all bootstrap samples for the respective experimental setup. Boxplots show Jaccard index values for each animal included in the bootstrap samples for female **(c)** and male **(f)** mice. The Jaccard index indicates the proportion of times an animal was assigned to its original cluster during the bootstrap sampling procedure. N phasic females = 89, n sustained females = 55, n phasic males = 105, n sustained males = 75


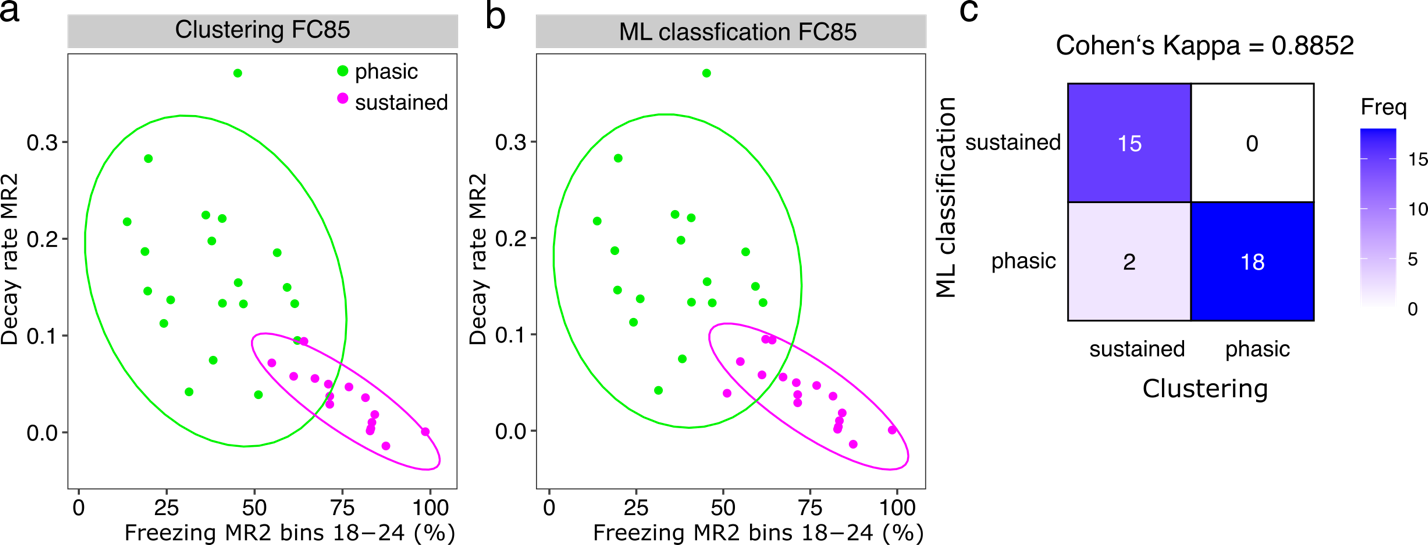


**Supplementary Figure 3. Performance of the ML classification models for the independent batch FC85 for memory retrieval (MR) 2. a)** Bivariate scatter plot showing Gaussian mixture model clustering results for the independent batch of female animals FC85 that was not included in training the supervised ML models. **b)** Bivariate plot showing classification of FC85 into sustained and phasic responders using the pre-trained ML model. Individual data points including 95% confidence ellipses are shown. **c)** Confusion matrix showing the concordance of clustering and ML classification results for FC85.

**Supplementary Table 1. Overview of animal batches included in the study.**

| **ML training batches** | | | |
| --- | --- | --- | --- |
| **Females** | | **Males** | |
| **MR1** | **MR2** | **MR1** | **MR2** |
| FC72 (n=24) | FC72 (n=24) | FC68 (n=27) | FC68 (n=27) |
| FC73 (n=24) | FC81 (n=40) | FC69 (n=16) | FC69 (n=16) |
| FC81 (n=40) | FC82 (n=40) | FC70_b (n=16) | FC70_b (n=16) |
| FC82 (n=40) | FC83 (n=40) | FC70_nb (n=17) | FC70_nb (n=17) |
| FC83 (n=40) |  | FC71 (n=20) | FC72 (n=24) |
|  |  | FC72 (n=24) | FC81 (n=40) |
|  |  | FC73 (n=24) | FC82 (n=40) |
|  |  | FC81 (n=40) |  |
|  |  | FC82 (n=40) |  |
|  |  |  |  |
| **Independent batches** | | | |
| **Females** | |  |  |
| **MR1** | **MR2** |  |  |
| FC85 (n=38) | FC85 (n=35) |  |  |
| FC87 (n=40) | FC87 (n=40) |  |  |

MR: Memory retrieval; FC: Fear conditioning

|  | **SVM** | **RSVM** | **Logistic regression** | **LDA** | **RandomForest** |
| --- | --- | --- | --- | --- | --- |
|  | Females MR1 | | | | |
| **Accuracy** | 0.938 ± 0.031 | 0.953 ± 0.028 | 0.966 ± 0.023 | 0.935 ± 0.033 | 0.926 ± 0.033 |
| **Sensitivity** | 0.927 ± 0.052 | 0.933 ± 0.049 | 0.975 ± 0.035 | 0.905 ± 0.06 | 0.927 ± 0.05 |
| **Specificity** | 0.953 ± 0.047 | 0.981 ± 0.032 | 0.955± 0.045 | 0.977 ± 0.037 | 0.928 ± 0.056 |
| **F1** | 0.942 ± 0.03 | 0.957 ± 0.027 | 0.968 ± 0.021 | 0.941 ± 0.031 | 0.931 ± 0.033 |
| **AUROC** | 0.937 ± 0.031 | 0.952 ± 0.029 | 0.965 ± 0.023 | 0.933 ± 0.033 | 0.926 ± 0.034 |
|  | Females MR2 | | | | |
| **Accuracy** | 0.971 ± 0.027 | 0.983 ± 0.018 | 0.989 ± 0.014 | 0.910 ± 0.04 | 0.979 ± 0.021 |
| **Sensitivity** | 0.954 ± 0.062 | 0.967 ± 0.046 | 0.997 ± 0.017 | 0.905 ± 0.069 | 0.962 ± 0.042 |
| **Specificity** | 0.983 ± 0.028 | 0.993 ± 0.016 | 0.985 ± 0.021 | 0.914 ± 0.052 | 0.989 ± 0.026 |
| **F1** | 0.962 ± 0.036 | 0.978 ± 0.025 | 0.986 ± 0.019 | 0.877 ± 0.057 | 0.971 ± 0.03 |
| **AUROC** | 0.973 ± 0.025 | 0.985 ± 0.016 | 0.991 ± 0.013 | 0.901 ± 0.046 | 0.98 ± 0.023 |
|  | Males MR1 | | | | |
| **Accuracy** | 0.901 ± 0.033 | 0.916 ± 0.032 | 0.918 ± 0.026 | 0.870 ± 0.039 | 0.918 ± 0.032 |
| **Sensitivity** | 0.913 ± 0.052 | 0.922 ± 0.058 | 0.93 ± 0.054 | 0.858 ± 0.074 | 0.906 ± 0.062 |
| **Specificity** | 0.896 ± 0.046 | 0.914 ± 0.048 | 0.911 ± 0.039 | 0.881 ± 0.057 | 0.928 ± 0.040 |
| **F1** | 0.866 ± 0.046 | 0.887 ± 0.044 | 0.897 ± 0.035 | 0.827 ± 0.054 | 0.893 ± 0.043 |
| **AUROC** | 0.889 ± 0.037 | 0.907 ± 0.036 | 0.921 ± 0.028 | 0.861 ± 0.042 | 0.913 ± 0.034 |
|  | Males MR2 | | | | |
| **Accuracy** | 0.976 ± 0.018 | 0.964 ± 0.023 | 0.973 ± 0.021 | 0.975 ± 0.024 | 0.955 ± 0.029 |
| **Sensitivity** | 0.964 ± 0.035 | 0.957 ± 0.042 | 0.978 ± 0.035 | 0.974 ± 0.034 | 0.953 ± 0.048 |
| **Specificity** | 0.985 ± 0.022 | 0.970 ± 0.034 | 0.97 ± 0.029 | 0.975 ± 0.038 | 0.957 ± 0.038 |
| **F1** | 0.971 ± 0.022 | 0.957 ± 0.028 | 0.967 ± 0.026 | 0.969 ± 0.028 | 0.944 ± 0.036 |
| **AUROC** | 0.977 ± 0.018 | 0.964 ± 0.024 | 0.974 ± 0.022 | 0.974 ± 0.024 | 0.953 ± 0.03 |

**Supplementary Table 2. Performance metrics for supervised ML models trained to classify female and male mice into sustained and phasic responders using freezing data from memory retrieval (MR) 1 and MR2.** Data are shown as mean ± standard deviation. SVM: support vector machine; RSVM: radial support vector machine; LDA: linear discriminant analysis. Model accuracies were calculated by subsetting the data in random training and test splits with a ratio of 70:30% over 1000 iterations. In each iteration, the model was trained on the training split; the respective accuracy was calculated on the test split.

**References**

1. Kovlyagina I, Wierczeiko A, Todorov H, Jacobi E, Tevosian M, von Engelhardt J, et al. Leveraging interindividual variability in threat conditioning of inbred mice to model trait anxiety. PLOS Biology. 2024;22(5):e3002642. doi: 10.1371/journal.pbio.3002642.
